# Comparative transcriptome profiling reveals the mechanism of increasing lysine and tryptophan content through pyramiding *opaque2*, *opaque16* and *waxy1* genes in maize

**DOI:** 10.1270/jsbbs.23051

**Published:** 2024-08-14

**Authors:** Peizhen Wu, Yanli Yuan, Zhoujie Ma, Kaiwu Zhang, Lei Deng, Hong Ren, Wenpeng Yang, Wei Wang

**Affiliations:** 1 Key Laboratory of Plant Resource Conservation and Germplasm Innovation in Mountainous Region (Ministry of Education), College of Life Sciences/Institute of Agro-bioengineering, Guizhou University, Guiyang 550025, Guizhou Province, China; 2 Guizhou Institute of Upland Food Crops, Guizhou Academy of Agricultural Sciences, Guiyang 550006, Guizhou Province, China; 3 Key Laboratory of Maize Biology and Genetic Breeding in Karst Mountainous Region (Ministry of Agriculture and Rural Affairs), Guiyang, 550006, Guizhou Province, China

**Keywords:** lysine, tryptophan, *o2*, *o16*, *wx*, gene pyramiding, differentially expressed genes

## Abstract

To explore the molecular mechanism behind maize grain quality and use of different gene stacking to improve the nutritional quality of grain, marker-assisted selection (MAS) was used to select three recessive mutant lines containing *o2o16wx*, along with the double-recessive mutant lines containing *o2o16*, *o2wx*, and *o16wx*. The resulting seeds were taken for transcriptome sequencing analysis 18 days after pollination (DAP). Results: Compared with the recurrent parent genes, in the lysine synthesis pathway, the gene pyramiding lines (*o2o16wx*, *o2wx*, and *o16wx*) revealed that the gene encoding aspartate kinase (AK) was up-regulated and promoted lysine synthesis. In the lysine degradation pathway, ‘QCL8010_1’ (*o2o16wx*) revealed that the gene encoding saccharopine dehydrogenase (LKR/SDH) was down-regulated. In addition, the gene pyramiding lines (*o2o16wx*, *o2o16*, and *o16wx*) indicated that the gene encoding 2-oxoglutarate dehydrogenase E1 component (OGDH) was down-regulated, inhibiting the degradation of lysine. In the tryptophan synthesis pathway, the genes encoding anthranilate synthase (AS), anthranilate synthase (APT), and tryptophan synthase (TS) were up-regulated (in *o2o16wx*, *o2o16*, *o2wx*, and *o16wx*), and promote tryptophan synthesis. In the tryptophan degradation pathway, it was revealed that the genes encoding indole-3-producing oxidase (IAAO) and indole-3-pyruvate monooxygenase (YUCCA) were down-regulated. These results provide a reference for revealing the mechanism of the *o2*, *o16*, and *wx* with different gene pyramiding to improve grain quality in maize.

## Introduction

Maize (*Zea mays* ssp. *mays* L.) is one of the three major food crops around the globe and represents the world’s highest total crop yield (Wang *et al.* 2022); it is also planting area is the maximum food crop in China (Zhang 2022). Corn kernels contain nutrients such as starch, protein, fat, vitamins, and minerals. The lysine and tryptophan in protein are essential amino acids for monogastric animals such as humans, livestock, and poultry. The contents of lysine and tryptophan in ordinary corn kernels are relatively low, which means that they cannot satisfy the needs of the human body for these amino acids (Ou 2016). Therefore, it is essential to increase the contents of lysine and tryptophan in corn kernels and improve the protein quality.

High-lysine maize has a higher level of lysine and higher tryptophan content than ordinary maize (Amegbor *et al.* 2022, Prasanna *et al.* 2001). A study by Lu (2004) found that the lysine and tryptophan contents in high-lysine maize were 140% and 117% higher than those in ordinary maize, respectively. Mertz *et al.* (1964) measured the amino acids of common maize and ‘*opaque-2*’ (*o2*) mutant maize via the copper extraction–fractionation method; the results showed that the lysine content of the *o2* mutant seed endosperm was more than 69% higher than that of common maize. The *o2* gene is one of the regulatory genes that inhibit the accumulation of zein in maize. It can increase the content of tryptophan and lysine by changing the expression of endosperm proteins such as amino acids via biosynthesis, starch, stress responses, and signal transduction, consequently, to improve the quality of maize (Huang *et al.* 2005, 2006, Prasanna *et al.* 2001, Soave *et al.* 1981).

Pukalenthy *et al.* (2020) introduced the *o2* gene into ordinary corn; the improved strain had better agronomic performance and the tryptophan content was increased by 0.040–0.048%. Subsequently, high-lysine mutants were found in other studies. Azevedo *et al.* (2004) compared the amino acid contents of ‘*opaque-5*’ (*o5*) and ‘*opaque-7*’ (*o7*) mutants, along with their corresponding wild-type materials. The soluble lysine concentration of the *o5* mutant was slightly reduced, while the soluble lysine concentration of the *o7* mutant was 15% higher. Yang (2005) and Yang *et al.* (2005) screened a new high lysine mutant from Robertson’s Mutator mutant library, named ‘*opaque-16*’ (*o16*). Further studies have shown that this gene has an additive effect on maize lysine content. Tian *et al.* (2022) identified a new mutation via ethylmethane sulphonate mutagenesis, named 3605K (dnj*-N1534, *o18-1*). Protein and amino acid detection showed that the lysine content in the resulting grains was 76.4% higher than that in the wild type. The discovery of these mutants provides a reference for breeding to improve maize quality.

The stacking of high-lysine mutant genes can further improve the nutritional quality of maize grains. Hartings *et al.* (2011) polymerized the *o2* and *o7* genes and found that the lysine content in the grains of the *o2o7* double-mutant maize was more than 3.5 times that of ordinary maize. Zhang (2010) used the ‘Taixi 19’ (*o2*) and ‘QCL3021’ (*o16*) lines as materials to breed *o2o16* double-mutant lines with a lysine content of 0.541%, which met the nutritional quality requirements for human consumption and food processing (Tian *et al.* 2004). Compared with the *o2* and *o16* single-mutant maize strains, this was an increase of 22.33% and 65.9%, respectively. Sarika *et al.* (2018a, 2018b) infiltrated *o16* into the parental inbred lines of *o2*, after which the average contents of lysine and tryptophan were 0.48% and 0.13%, respectively. Compared with the contents in the original parents, the average contents of lysine and tryptophan in the offspring increased by 49% and 60%, respectively (Sarika *et al.* 2018a, 2018b).

‘*Waxyl*’ (*wx*) maize is a spontaneous mutation of common maize, which is inherited via the recessive single gene, *wx*. The amylopectin in waxy maize endosperm is 95%–100%, and its unique flavor is loved by most people (Shi *et al.* 2001). In order to obtain waxy maize with a good flavor and high lysine content, Zhang *et al.* (2009) used marker-assisted selection (MAS) to breed high-quality protein waxy maize (*wx*-QPM) inbred lines, where the lysine content was between 0.36% and 0.54%. Talukder *et al.* (2022, 2023) created the *o2wx* mutant germplasm, in which the lysine and tryptophan contents reached a peak at 20 days after pollination (DAP), recorded at 0.552% and 0.267%, respectively. Wang *et al.* (2019b) used conventional breeding techniques combined with MAS to breed *o16wx* near-isogenic lines. Compared with ordinary maize, lysine content increased by 34.4%. Chen (2008) obtained a single plant of the *o2o16wx* genotype by MAS, and the average lysine content of the selected single plant was 0.462%. Zhang (2010) selected the *o2o16wx* mutant line by molecular breeding, and the average lysine content was 0.616%, which was 94.3% higher than that of *wx* maize.

In order to study the molecular mechanism behind maize quality and the use of gene stacking to improve nutritional quality, Hartings *et al.* (2011) selected the *o2o7* double-mutant maize strain for transcriptome sequencing. Compared with the wild-type tryptophan synthesis pathway, the *o2*o7 double-mutant anthranilate phosphoribosyl transferase (APT) [EC:2.4.2.18] and o-aminobenzoic acid synthase [EC:4.1.3.27] were up-regulated and promoted tryptophan synthesis. The lysine content of the *o2wx* mutant line selected for analysis by Wang *et al.* (2019a) was higher than that of the *wx* line, and the 18 DAP sample was subjected to RNA sequencing (RNA-Seq). The results showed that the differentially expressed genes (DEGs) of the *o2wx* mutant lines were mainly related to catalytic activity and metabolic processes. The two genes encoding EF-1α and LHT1 were up-regulated, but the genes encoding sulfur-rich proteins were down-regulated, thereby increasing the lysine content. Zhou *et al.* (2016) successfully improved the lysine content and quality of waxy maize by using MAS to infiltrate the *o2* mutant into waxy maize lines. Proteomics analysis showed that *o2* gene introgression reduced the accumulation of zein and even affected other endosperm proteins that are related to amino acid biosynthesis and the starch–protein balance. However, the ways in which the different stacking types of the high-lysine mutant genes *o2*, *o16*, and *wx* in maize improve the nutritional quality of grain have not been reported.

Based on this, using ‘CML530’ as the receptor and QCL8002_11 (*o2o16wx*) (Zhang 2010, Zhang *et al.* 2013) as the donor, we bred *o2o16wx* triple-recessive and *o2o16*, *o2wx*, and *o16wx* double-recessive gene pyramiding lines containing the *o2*, *o16*, and *wx* genes by MAS=. ‘CML530’ is the maize germplasm of acid soil tolerance, which derived from the International Maize and Wheat Improvement Center (CIMMYT) and adapted to South America. Subsequently, RNA-Seq was performed at the grain development stage (18DAP) to compare the transcriptional expression differences between the gene pyramiding lines and their parents, in order to understand the molecular mechanism and different gene stacking of the *o2*, *o16*, and *wx* genes on the nutritional quality of maize grains.

## Materials and Methods

### Plant materials

Using ‘QCL8002_11’ (*o2o16wx*) as the donor and the common maize inbred line ‘CML530’ (wild-type, WT) as the receptor, F_1_ was obtained via hybridization. In the F_1_ generation, BC_1_F_1_ was obtained via backcrossing with ‘CML530’. In the BC_1_F_1_ generation, individuals with the genotype *O2o2O16o16wxwx* were selected, for *o2* and *wx* were carried out using gene-based SSR markers phi112 (Danson *et al.* 2010, Yang *et al.* 2004) and phi061 (Li *et al.* 2002) respectively, and for *o16*, linked markers, umc1141 was used (Yang *et al.* 2005) (Supplemental Table 1). BC_2_F_1_ was obtained by backcrossing with ‘CML530’. In the BC_2_F_1_ generation, individual plants with the genotype of *O2o2O16o16WXwx* were selected and self-cross to obtain BC_2_F_2_. In the BC_2_F_2_ generation, individual plants with genotypes of *o2o16wx*, *o2o16*, *o2wx*, and *o16wx* were selected, and BC_2_F_3_ was obtained by self-cross. Combined with a genetic background analysis, lysine content analysis, and the field performance of the resulting plants, four different types of gene pyramiding materials were finally selected (Supplemental Fig. 1). After multiple generations of self-cross, the plants were genetically stable and were named ‘QCL8010_1’ (*o2o2o16o16wxwx*, *o2o16wx*), ‘QCL8010_4’ (*o2o2o16o16WXWX*, *o2o16*), ‘QCL8010_5’ (*o2o2O16O16wxwx*, *o2wx*), and ‘QCL8010_6’ (*O2O2o16o16wxwx*, *o16wx*). The SNP (55K) chip analysis showed that the genetic background recovery rates of the *o2*, *o16*, and *wx* different combination types of gene pyramiding lines, based on molecular markers, were 93.17%, 92.52%, 92.40%, and 93.23%, respectively; these percentages were higher than the theoretical background recovery rate of 87.50%. In 2017, different combinations of *o2*, *o16*, and *wx* were planted in the experimental field of the Guizhou Academy of Agricultural Sciences (Guiyang, China) (N26°30ʹ14ʺ, E106°39ʹ21ʺ). Three ear rows per line were planted with sixty plants total.

### Grain characteristics and submicroscopic structure

The mature and dry seeds from the *o2*, *o16*, and *wx* different combination types of gene pyramiding lines and their recurrent parent, ‘CML530’, were selected to observe the ear of maize under natural light, and the seeds were placed on a seed purity worktable (TJD_1300A, Zhejiang) to determine its transparency. The grain was peeled off with a blade to observe the degree of flour and horniness throughout the grain section and was then photographed with a camera. The grain phenotype and the 100-grain weight were measured using the Wanshen seed phenotype and appearance quality analyzer (SG_G, Hangzhou). Corn kernels were cut transversely with a blade, and a small amount of iodine droplets were added to the cross section of the kernels to observe the color change. Variance analysis was performed using the IBM SPSS Statistics 25 program (the same program is also referenced below).

After removing the peel of the mature and dry kernels of maize, a small piece of endosperm was cut from the middle of the kernel using a blade, and the platinum was embedded via ion sputtering with an E_1010 ion sputtering instrument (E_1010, HITACHI, Japan). Observations were then carried out using a scanning electron microscope (SEM, S_3400N). Observation of the submicroscopic structure was completed by the Guizhou Provincial Key Laboratory of Agricultural Biotechnology (Guizhou Academy of Agricultural Sciences, Guiyang, Guizhou).

### Grain quality and amino acid analysis

Near-infrared spectroscopy (NIRSTM DS2500, Guangdong) was used to analyze the quality of the mature maize kernels, including the contents of oil, fat, protein, and starch in the samples. The instrument measurement parameters were set up. The sample cups were loaded with maize kernels and put inside the instrument for testing. Each sample was repeated twice, and the average value was taken.

The amino acid content of the mature maize kernels was analyzed with an automatic amino acid analyzer (S_433D, Sykam, Germany). After the corn kernels were dried, they were crushed using a universal pulverizer (FW100, Taisite, Tianjin). The pulverized kernels were then passed through an 80-mesh sieve and weighed into 120–150 mg samples, which were placed in a 15 mL hydrolysis tube (with a screw port), 5 mL of 6 mol/L HCl was added, and the samples were water-bathed at 110 °C for 20–24 h, cooled to room temperature, adjusted to a pH of 2.0 with 6 mol/L NaOH solution, and finally diluted to 100 mL. The reaction solution was absorbed using a syringe. The sample was filtered through a 45 μm water filter membrane into a 2 mL liquid chromatography injection bottle (Ningbo Hamai Instrument Technology Co., Ltd.), and the contents of 17 free amino acids were detected by the machine. The testing of each sample was repeated 2 times and the average value was taken.

Tryptophan content was determined according to the national standard, GB7850-87, and the testing of each sample was repeated three times. L_tryptophan (BWJ4259-2016) was selected as the standard substance and the absorbance was measured at 590 nm using an ultraviolet-visible spectrophotometer (754N, Shanghai). The standard curve was drawn, and the corresponding tryptophan content of each sample was calculated.

### RNA library construction and sequencing

A minimum of three well-filled ears of each genotype were sampled at 18DAP, and grinded in liquid nitrogen to form a powder. Total RNA of the whole grains was extracted using a Plant RNA Kit (R6827_01, OMEGA). The concentration and quality of the RNA were detected using an Agilent 2100 bioanalyzer (Agilent RNA 6000 Nano Kit). Oligo (dT) magnetic beads were used to select mRNA with polyA tail. Fragment the target RNA and reverse transcription to double-strand cDNA by N6 random primer. The AMPure XP beads nucleic acid purification kit was utilized for the repair and ligation of synthetic cDNA. Subsequently, the linked products were amplified via PCR using specific primers, followed the single strand DNA is cyclized by splint oligo and DNA ligase to generate the cDNA library. Sequencing was performed using the BGISEQ_500 platform by the Shenzhen Huada Gene Technology Service Co., Ltd., and the testing of each sample was repeated three times.

### RNA-sequencing quality detection

The raw data obtained via sequencing were subjected to quality control. Clean reads were obtained after removing the joints (unknown bases are more than 10%) and low-quality raw reads (the percentage of low-quality bases is over 50% in a read; we define the low quality base to be the base whose sequencing quality is no more than 5). The filtered data were compared with the reference sequence (B73 Ref Gen_v4). Quality control of the sample data was carried out and the value of the QC content was calculated. RSEM (v1.2.12) (Li and Dewey 2011) was used to quantify the gene expression, and the number and proportion of genes identified in each sample were counted according to the results. Fragments Per Kilobase of exon model per Million mapped fragments (FPKM) method is used in calculated expression level (Florea *et al.* 2013). According to the quantitative results, the correlation between each two samples of all the samples was calculated, and a correlation heat map was obtained.

### Gene ontology (GO) and Kyoto encyclopedia of genes and genomes (KEGG) analysis of DEGs

The NOISeq software was used to calculate the fold difference of each gene (Tarazona *et al.* 2011), then the DEGs were screened out (foldchange ≥2 and diverge probability ≥0.8). Cluster software was used to cluster the DEGs via the Euclidean distance (Eisen *et al.* 1998). GO enrichment analysis was performed on the selected DEGs, then GO functional classification statistics were performed on the DEGs using the WEGO software (Ye *et al.* 2006). Pathway analysis and the functional annotation of DEGs were performed using the KEGG database output (Kanehisa *et al.* 2008).

### qRT-PCR Analysis

Nine DEGs were selected for qRT-PCR verification, and primer design was performed using a website (https://www.primer3plus.com/cgi-bin/dev/primer3plus.cgi) (Supplemental Table 2). The cDNA was synthesized using a reverse transcription kit (Thermo Scientific Revert Aid First Stand cDNA Synthesis Kit, K1622). Quantitative analysis was performed using the SYBR^®^ Select Master Mix kit (Applied Biosystems Inc., California, USA) on the CFX Connect real-time PCR system (BIO-RAD). The testing of each sample was repeated three times, and Actin 1 (https://www.maizegdb.org) of maize was used as an internal reference. The relative expression of the gene was calculated according to the 2^–ΔΔCT^ value.

### Accession numbers

The raw data of RNA-Seq have been submitted to https://ngdc.cncb.ac.cn/. CRA011451records will be accessible upon publication on the indicated release date.

## Results

### Grain characteristics

**Fig. 1.**
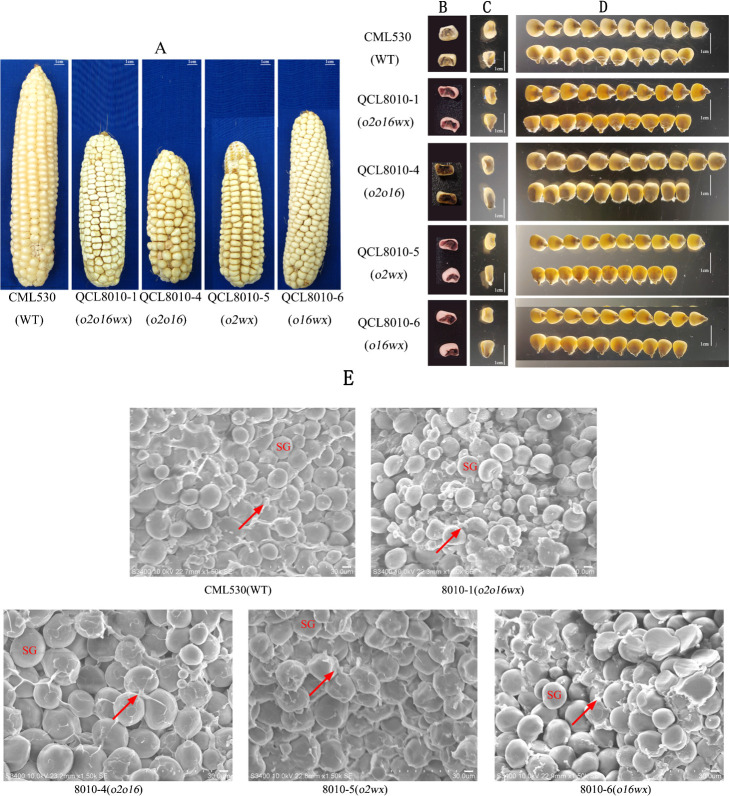
The phenotypes of kernels and electron micrographs of endosperm of WT and gene pyramiding lines (*o2o16wx*, *o2o16*, *o2wx*, *o16wx*). (A) Mature ear with WT and gene pyramiding lines; (B) Starch staining kernels phenotype; (C) Transparency of kernels cross section under transmitted light; (D) Kernels transparency under projected ligh; (E) The magnification is 1500 times; SG represents starch granules; the arrow refers to the matrix protein.

The phenotypes of the gene pyramiding lines and their recurrent parents were observed under natural light, while the transparency and the grain’s longitudinal and transverse sections were observed under projected light (Fig. 1). In the recurrent parent ‘CML530’, the seed coat is smooth and glossy, the grain is full, the endosperm is horned, and the grain is transparent. The ‘QCL8010_1’ (*o2o16wx*) mutant line is of the ear tube type; the grain is dull, with a powdery endosperm, and the grain is opaque. The ‘QCL8010_4’ (*o2o16*) mutant line is of the ear tube type; the grain is slightly larger and has a dull, powdery endosperm, and the grain is basically opaque. The ‘QCL8010_5’ (*o2wx*) mutant line is of the ear tube type; the grain is dull, with a silty endosperm. The ‘QCL8010_6’ (*o16wx*) mutant line is of the ear cylinder type, with a waxy endosperm. The samples were stained with endosperm starch. ‘CML530’ and ‘QCL8010_4’ (*o2o16*) appears blue. ‘QCL8010_1’ (*o2o16wx*), ‘QCL8010_5’ (*o2wx*) and ‘QCL8010_6’ (*o16wx*) are purple-red.

When viewed under the scanning electron microscope (SEM), compared with ‘CML530’, the starch granules of ‘QCL8010_1’ (*o2o16wx*) and ‘QCL8010_6’ (*o16wx*) were mostly oval and relatively smooth, and the matrix protein density was higher, being dispersed in the starch granules. The starch granules of ‘QCL8010_4’ (*o2o16*) were slightly larger, while the starch granules of ‘QCL8010_5’ (*o2wx*) were irregular (Fig. 1D). According to the measurement results from the Wanshen seed phenotype and appearance quality analyzer (SG_G, Hangzhou), compared with the recurrent parent ‘CML530’, there was no significant change in the 100-kernel weight, kernel length, and kernel width of the gene pyramiding lines in the different combinations of *o2*, *o16*, and *wx* (Supplemental Table 3).

### Quality analysis

**Fig. 2.**
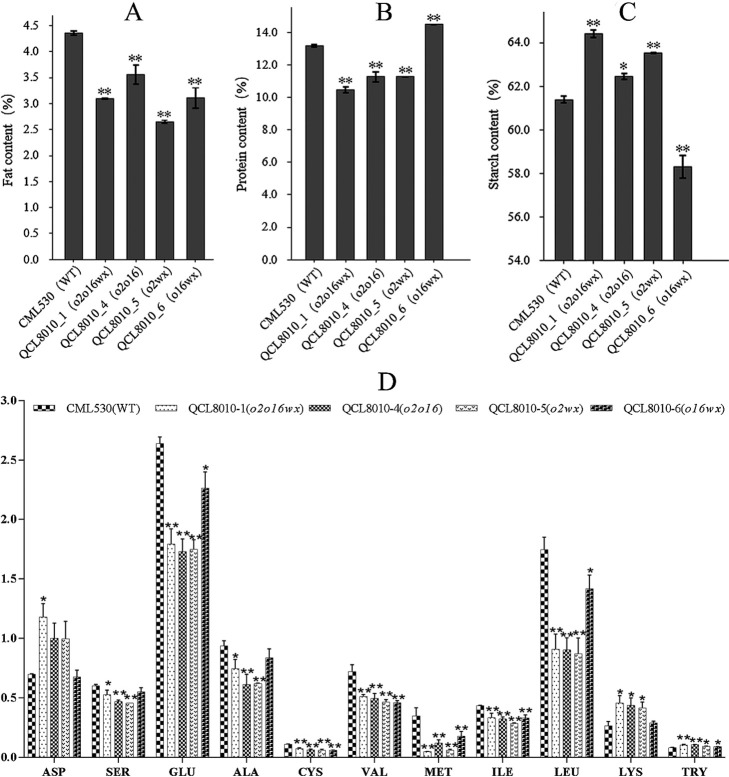
Percentage of quality content of WT and gene pyramiding lines (*o2o16wx*, *o2o16*, *o2wx*, *o16wx*). (A) The oil content of maize kernels; (B) Protein content of maize kernels; (C) Starch content in maize kernels; (D) Contents of amino acids in mature kernels. * is indicated as a significant change; ** is indicated as a very significant change compared to the recurrent parent CML530. The error line is SD.

Compared with the recurrent parent ‘CML530’, the oil content and protein content of the *o2*, *o16*, and *wx* gene pyramiding lines were significantly reduced, and the protein content of gene pyramiding lines (*o2o16wx*, *o2o16*, *o2wx*) was reduced by 20.57%, 14.42%, and 14.38%, respectively. The starch content of gene pyramiding lines (*o2o16wx*, *o2o16*, *o2wx*) increased slightly by 4.92%, 1.73%, and 3.48%, respectively. The oil and starch contents of ‘QCL8010_6’ (*o16wx*) decreased slightly and the protein content increased by 10.06% (Fig. 2).

Compared with the recurrent parent ‘CML530’, the Glu, Cys, Val, Met, Ile, and Leu contents of the different combinations of the *o2*, *o16*, and *wx* gene pyramiding lines decreased (Fig. 2D). The contents of Ser and Ala in the gene pyramiding lines (*o2o16wx*, *o2o16*, and *o2wx*) decreased. The Lys content of the gene pyramiding lines (*o2o16wx*, *o2o16*, and *o2wx*) increased significantly by 72.07%, 65.61%, and 56.67%. There was no significant change in the Lys content of ‘QCL8010_6’ (*o16wx*). The Try content of the different combinations of the *o2*, *o16*, and *wx* gene pyramiding lines increased by 30.00%, 32.50%, 15.00% and 19.6%. The remaining amino acids did not change significantly.

### Correlation between RNA-seq quality and biological repeats

According to the sequencing results, an average of 24,028,098 clean reads were obtained from 15 samples (Supplemental Table 4). The average comparison rate with the reference gene (B73) was 82.31% (Supplemental Table 5), while the average comparison rate with the reference genome was 91.10%. The proportion of bases with a quality value (Q20) ≥20 was greater than 97%, indicating that the quality control of the sequencing data was good and could be used for further analysis (Supplemental Table 6). According to the results of the quantitative analysis, the average number of genes detected in the 15 samples accounted for more than 71% of the total number of genes 39,498 (Supplemental Fig. 2), and the sequencing saturation was in line with expectations. The average correlation coefficient between the three replicates of the sample (Supplemental Fig. 3) was 0.985, which was greater than 0.96, indicating that the RNA-seq sequencing quality was reliable and could be used for quantitative analysis.

### Identification of DEGs

**Fig. 3.**
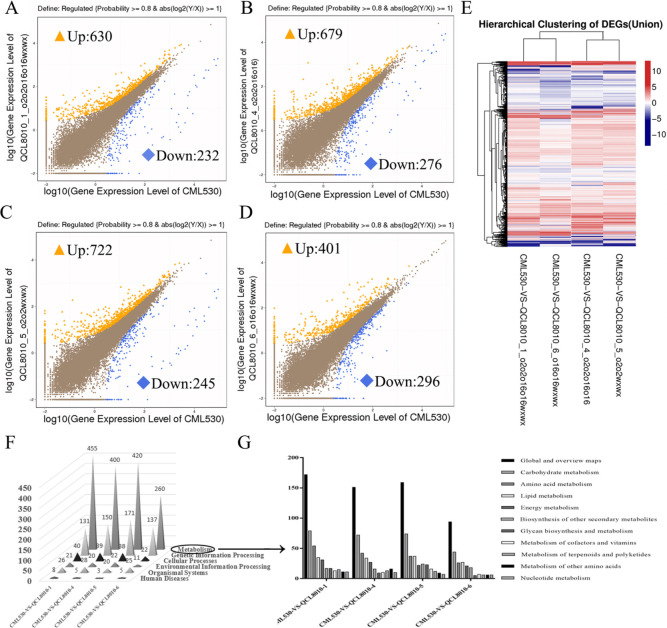
Conduct KEGG pathway enrichment analysis and hierarchical clustering analysis on DEGs of WT and gene aggregation lines (*o2o16wx*, *o2o16*, *o2wx*, *o16wx*) at 18DAP. (A–D) The results of differential analysis between ‘QCL8010_1’, ‘QCL8010_4’, ‘QCL8010_5’, ‘QCL8010_6’ and recurrent parent ‘CML530’; (E) Hierarchical cluster analysis of samples, each column represents a difference pair, each row represents a differential gene, different types of differential genes are expressed in different colors, red indicates up-regulation, blue indicates down-regulation, and the degree of up-regulation and down-regulation increases with the deepening of color; (F) KEGG pathway DEGs; (G) Metabolism pathway DEGs.

The NOISeq software was used to analyze the DEGs of the tested samples, while cluster software was used to cluster the DEGs (Fig. 3). Compared with the recurrent parent ‘CML530’, ‘QCL8010_1’ (*o2o16wx*) had 862 DEGs, of which 630 were up-regulated and 232 were down-regulated (Fig. 3A); ‘QCL8010_4’ (*o2o16*) had 955 DEGs, of which 679 were up-regulated and 276 were down-regulated (Fig. 3B); ‘QCL8010_5’ (*o2wx*) had 967 DEGs, of which 722 were up-regulated and 245 were down-regulated (Fig. 3C); ‘QCL8010_6’ (*o16wx*) had 697 DEGs, of which 401 were up-regulated and 296 were down-regulated (Fig. 3D).

### Functional annotation of DEGs

In order to understand the function of DEGs, WEGO software was used to perform GO functional classification statistics analysis on the obtained DEGs. Compared with the recurrent parent ‘CML530’, ‘QCL8010_1’ (*o2o16wx*) incorporated 2391 DEGs, involving 36 GO terms. Regarding the biological process (BP), 254 DEG genes were involved in the metabolic process. Regarding molecular function (MF), 269 DEG genes were involved in catalytic activity. ‘QCL8010_4’ (*o2o16*) exhibited 2560 DEGs involving 35 GO terms, 270 DEGs that were involved in the metabolic process in terms of BP, and 258 DEGs that were involved in catalytic activity in terms of MF. ‘QCL8010_5’ (*o2wx*) exhibited 2655 DEGs involving 34 GO terms. Regarding BP, 282 DEG genes were involved in the metabolic process. Regarding MF, 282 DEGs genes were involved in catalytic activity. In ‘QCL8010_6’ (*o16wx*), 2029 DEGs involved 37 GO terms, 203 DEGs involved the metabolic process in terms of BP, and 190 DEGs involved catalytic activity in terms of MF (Supplemental Fig. 4).

The functional annotation of DEGs was performed using the KEGG public database output (Fig. 3F). Compared with the recurrent parent ‘CML530’, the DEGs were annotated into 20 pathways, most of which were annotated to metabolism (Fig. 3G). Further analysis of the metabolism pathway revealed that most of the DEGs were enriched in both global and overview maps for carbohydrate metabolism and amino acid metabolism. Among them, regarding amino acid metabolism, 9, 5, 6, and 4 DEGs of ‘QCL8010_1’ (*o2o16wx*), ‘QCL8010_4’ (*o2o16*), ‘QCL8010_5’ (*o2wx*), and ‘QCL8010_6’ (*o16wx*) were annotated to the plant lysine metabolic pathway, while 4, 5, 4, and 1 DEGs were annotated to the plant tryptophan metabolic pathway, respectively. At the same time, 17, 16, 15, and 1 gliadin genes were detected in the gene stacking materials for the same lines, respectively.

### DEGs in lysine metabolism

**Fig. 4.**
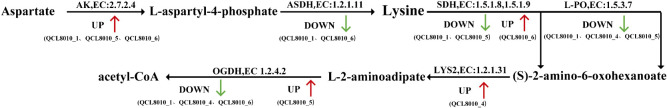
Schematic representation of the Lysine metabolic pathway in maize. In the diagram, UP represents the upregulation of the gene encoding the enzyme, while DOWN represents the downregulation of the gene encoding the enzyme. The sample name represents a change in the gene encoding the enzyme in the reaction. AK: aspartate kinase; ASDH: aspartate-semialdehyde dehydrogenase; L-PO: L-pipecolate oxidase; LKR/SDH: saccharopine dehydrogenase; LYS2: L-aminoadipate-semialdehyde dehydrogenase; OGDH: 2-oxoglutarate dehydrogenase E1 component.

According to the results of the KEGG annotation, the DEGs in the lysine metabolic pathway of maize were analyzed (Fig. 4). Compared with the recurrent parent ‘CML530’, a total of 7 DEGs were detected in the maize gene stacking material, which were annotated to lysine metabolism. In total, 2 DEGs were positioned in the synthesis pathway and 5 DEGs were in the degradation pathway.

In the lysine synthesis pathway, the up-regulated expression of the gene *Zm00001d052656* encoding aspartate kinase (AK) [EC:2.7.2.4] was detected in ‘QCL8010_1’ (*o2o16wx*) and ‘QCL8010_6’ (*o16wx*), which promoted lysine synthesis; conversely, the gene *Zm00001d009968* encoding aspartate_semialdehyde dehydrogenase (ASDH) [EC:1.2.1.11] was down-regulated and inhibited lysine synthesis. AK [*Zm00001d052656*, EC:2.7.2.4] was up-regulated in ‘QCL8010_5’ (*o2wx*) to promote lysine synthesis. In the lysine degradation pathway, ‘QCL8010_1’ (*o2o16wx*) and ‘QCL8010_5’ (*o2wx*) detected that the gene *Zm00001d052079* encoding saccharopine dehydrogenase (LKR/SDH) [EC:1.5.1.8,1.5.1.9] and the gene *Zm00001d020984* encoding L-pipecolate oxidase (L-PO) [EC:1.5.3.7] were down-regulated and inhibited lysine degradation. ‘QCL8010_4’ (*o2o16*) detected that L-PO [*Zm00001d020984*, EC:1.5.3.7] was down-regulated and inhibited lysine degradation. The gene pyramiding lines (*o2o16wx*, *o2o16*, *o16wx*) detected that the gene *Zm00001d023552* encoding 2-oxoglutarate dehydrogenase E1 component (OGDH) [EC:1.2.4.2] was down-regulated and promoted lysine degradation.

In addition, compared with the recurrent parent ‘CML530’, a total of 18 DEGs were detected as being annotated for zein metabolism in different combinations of the *o2*, *o16*, and *wx* gene pyramiding lines (Supplemental Table 7), and most of the DEGs were down-regulated.

### DEGs in tryptophan metabolism

**Fig. 5.**
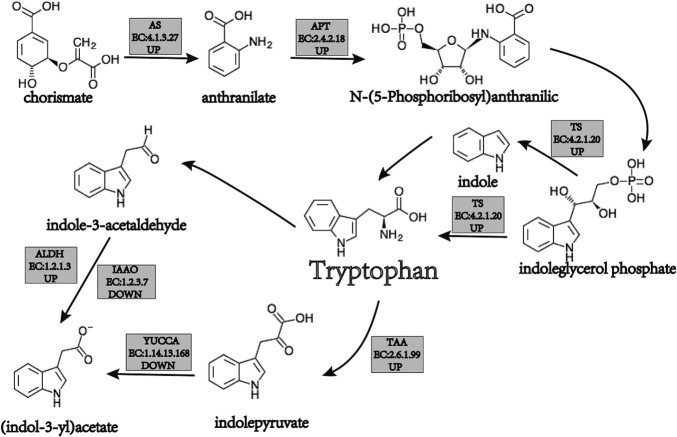
Schematic representation of the tryptophan metabolism pathway in maize. In the diagram, UP represents the upregulation of the gene encoding the enzyme, while DOWN represents the downregulation of the gene encoding the enzyme. AS: anthranilate synthase; APT: anthranilate synthase anthranilate synthase; TS: tryptophan synthase; TAA: L-tryptophan-pyruvate aminotransferase; ALDH: aldehyde dehydrogenase; IAAO: indole-3-producing oxidase; YUCCA: indole-3-pyruvate monooxygenase.

According to the results of the KEGG functional annotation, compared with the recurrent parent ‘CML530’, a total of 8 DEGs were annotated to the tryptophan metabolism pathway in the maize gene stacking material. In total, 4 DEGs were positioned in the synthesis pathway and 4 DEGs were positioned in the degradation pathway (Fig. 5).

Among them, in the tryptophan synthesis pathway, the *Zm00001d034713* encoding anthranilate synthase (AS) [EC:4.1.3.27], the *Zm00001d021606* and *Zm00001d030185* encoding anthranilate synthase anthranilate synthase (APT) [EC:2.4.2.18], and the *Zm00001d034452* encoding tryptophan synthase (TS) [EC:4.2.1.20] were up-regulated in the gene pyramiding lines (*o2o16wx*, *o2o16*, and *o2wx*). To promote tryptophan synthesis, ‘QCL8010_6’ (*o16wx*) was up-regulated in terms of TS [*Zm00001d034452*, EC:4.2.1.20] and promoted tryptophan synthesis. In the tryptophan degradation pathway, the gene pyramiding lines (*o2o16wx*, *o2o16*, and *o2wx*) detected that the gene *Zm00001d037498* encoding L-tryptophan-pyruvate aminotransferase (TAA) [EC:2.6.1.99] was up-regulated and promoted tryptophan degradation. ‘QCL8010_4’ (*o2o16*) and ‘QCL8010_5’ (*o2wx*) detected that the gene *Zm00001d023718* encoding indole-3-pyruvate monooxygenase (YUCCA) [EC:1.14.13.168] was down-regulated and inhibited tryptophan degradation. ‘QCL8010_4’ (*o2o16*) detected that the gene *Zm00001d003983* encoding aldehyde dehydrogenase (NAD+) (ALDH) [EC:1.2.1.3] was up-regulated and promoted tryptophan degradation. The gene *Zm00001d034388* encoding indole-3-producing oxidase (IAAO) [EC:1.2.3.7] was down-regulated and inhibited tryptophan degradation.

### DEGs in starch biosynthesis pathway

**Fig. 6.**
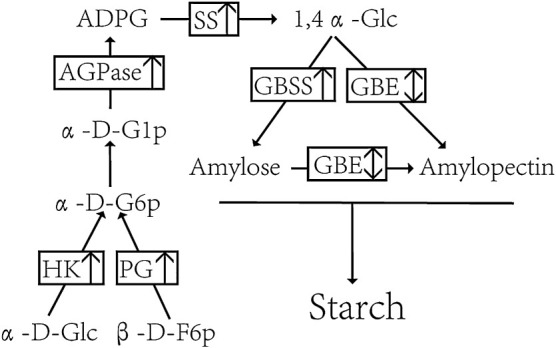
Schematic representation of the starch metabolism pathway in maize. The arrow upwards indicates an increase, and the double arrows indicate a combination of up-regulation and down-regulation. HK: hexokinase; PG: glucose-6-phosphate isomerase; AGPase: glucose-1-phosphate adenylyltransferase; SS: starch synthase; GBSS: granule-bound starch synthase; GBE: 4-alpha-glucan branching; α-D-Glc: α-D-glucose; β-D-F6p: β-D-Fructose-6p; α-D-G6P: α-D glucose-6-phosphate; α-D-G1P: α-D glucose-1-phosphate; ADPG: ADP-glucose; 1,4α-Glc: 1,4α-glucose.

According to the KEGG annotation results, a total of 7 DEGs were annotated to the starch synthesis pathway in the maize gene stacking material, compared with those in the recurrent parent during the starch metabolism process (Fig. 6).

Compared with the recurrent parent ‘CML530’, the gene *Zm00001d039512* encoding hexokinase (HK) [EC:2.7.1.1], the gene *Zm00001d050032*, the gene *Zm00001d032385*, and the gene *Zm00001d044129* encoding glucose-1-phosphate adenylyltransferase (AGPase) [EC:2.7.7.27] were detected in the gene pyramiding lines (*o2o16wx*, *o2o16*, and *o2wx*) was up-regulated and promoted starch synthesis. ‘QCL8010_1’ (*o2o16wx*) and ‘QCL8010_5’ (*o2wx*) detected genes *Zm00001d034256* encoding glucose-6-phosphate isomerase (PG) [EC:5.3.1.9] and genes *Zm00001d000002*, *Zm00001d045261* encoding starch synthase (SS) [EC:2.4.1.21], and encoding the gene *Zm00001d016684* of 4-alpha-glucan branching enzyme (GBE) [EC:2.4.1.18] was up-regulated to promote amylopectin synthesis. ‘QCL8010_4’ (*o2o16*) detected that the gene *Zm00001d029360* encoding granule-bound starch synthase (GBSS) [EC:2.4.1.242] was up-regulated and promoted amylose synthesis.

### qRT-PCR validation

**Fig. 7.**
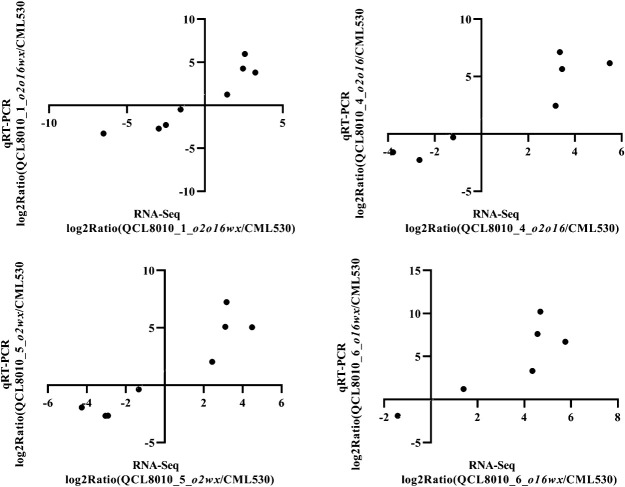
The qRT-PCR validation of 9 DGEs by RNA-seq.

In order to verify the results of the transcriptome sequencing, 9 significantly DEGs were randomly selected and verified using qRT-PCR. The results showed that in ‘QCL8010_1’ (*o2o16wx*)/‘CML530’, ‘QCL8010_4’ (*o2o16*)/‘CML530’, ‘QCL8010_5’ (*o2wx*)/‘CML530’, and ‘QCL8010_6’ (*o16wx*)/‘CML530’, there were 8, 7, 8, and 6 DEGs in which the qRT-PCR expression patterns were similar to the RNA-seq results, respectively. The Pearson coefficients of the RNA-seq and qRT-PCR results for the same genes were 0.8496, 0.8569, 0.8688, and 0.7523, respectively (Fig. 7).

## Discussion

To understand the molecular mechanism of *o2*, *o16*, and *wx* gene pyramiding and improve the nutritional quality of maize kernels, MAS was used to breed different combinations of the *o2*, *o16*, and *wx* gene pyramiding lines. The SNP (55K) chip analysis showed that the recovery rate of the genetic background of the mutant lines, based on the molecular markers, was more than 92%. Based on this finding, transcriptome sequencing analysis was performed at the grain development stage (18DAP). The results showed that the DEGs were mainly annotated to the metabolism of biological processes and to the catalytic activity of molecular functions. Significantly DEGs were detected in lysine, tryptophan, and starch metabolism, which provided a reference for elucidating the mechanism of *o2*, *o16*, and *wx* regarding different gene stacking to improve the nutritional quality of grain.

Lysine biosynthesis in organisms is mainly affected by two key enzymes, AK [EC:2.7.2.4] and dihydropicolinate synthase (DHPS) [EC:4.2.1.52] (Perl *et al.* 1992). AK [EC:2.7.2.4] catalyzes the first reaction in the biosynthesis pathway of aspartic acid-derived amino acids in plants and plays a key regulatory role in the lysine metabolic pathway of maize (Dotson *et al.* 1990, Gaziola *et al.* 1999). Wang *et al.* (2019b, 2019c) showed that the transcription level of AK was no significant change after introgression *o2* and *o16* alleles into waxy corn. However, we found that the gene encoding AK in the lysine biosynthesis pathway was up-regulated in triple recessive mutant (*o2o16wx*) and double recessive mutant (*o2wx*, *o16wx*), and promoted lysine synthesis.

In the process of lysine degradation, LKR/SDH is the first enzyme in this pathway, and participates in lysine catabolism, using NADPH as a cofactor to decompose lysine into saccharopine and (S)2-aminoadipate-6-semialdehyde (Goncalves-Butruille *et al.* 1996). In the mutation of *o2*, the transcription level of LKR/SDH mRNA decreased by more than 90%, thereby reducing the degradation of lysine (Wang *et al.* 2019b). Wang *et al.* (2019b) showed that the transcription level of LKR/SDH was down-regulated after introgression *o2* and *o16* alleles into waxy corn. Identically, in this study, we found that *lkrsdh1* encoding LKR/SDH [EC:1.5.1.8,1.5.1.9] was down-regulated at 18DAP in ‘QCL8010_1’ (*o2o16wx*) and ‘QCL8010_5’ (*o2wx*), and inhibited the degradation. Lysine degradation metabolism is not only affected by LKR/SDH, but also by L-PO. L-PO is the key enzyme in the transformation of lysine into 2-aminoadipate-6-semialdehyde via 6-amino-2-oxohexanoate and L-pipecolate. Wang *et al.* (2019a) performed RNA sequencing on the double-recessive mutant line *o2o16*, found that the gene encoding L-PO in the lysine degradation pathway was down-regulated. In this study, several gene stacking lines (*o2o16wx*, *o2o16*, and *o2wx*) detected the down-regulated expression of genes encoding L-PO and inhibited lysine degradation. OGDH can convert 2-oxoadipate into S-glutaryl-dihydrolipoamide. Studies have shown that OGDH is observed to control respiration in plant and animal systems, which, in some cases, leads to a decrease in the total amino acid content and lysine content in organisms (Araújo *et al.* 2012, Bunik and Fernie 2009). In this study, the gene encoding OGDH in the gene pyramiding lines (*o2o16wx*, *o2o16*, and *o16wx*) was down-regulated and inhibited lysine degradation.

Zein is the main storage protein of maize, accounting for about 45–50% of the maize’s protein (Shukla and Cheryan 2001). Zein-Alpha A20 (*Zm00001d019155* and *Zm00001d019156*) is closely related to the formation of powdery endosperm (Li *et al.* 2022). Studies have shown that maize germplasms with high lysine and tryptophan contents have lower zein contents (Gibbon and Larkins 2005). The *o2o16* mutant line exhibits medium levels of 19_kDa and 22_kDa α-zein (Sarika *et al.* 2018a). The lysine synthesis mechanism in the *o16* mutant is completely different from that of *o2*, while the *o2o16* mutant line has a higher lysine content than the *o2o2* mutant line alone (Yang 2005). In this study, we found that the Zein-Alpha A20 and 22_kDa α-zein genes (*Zm00001d019155* and *Zm00001d019156*) were down-regulated during grain development and inhibited gliadin synthesis in the gene pyramiding lines with different combinations of the materials *o2*, *o16*, and *wx*.

There are 6 steps that comprise enzymatic reactions in plant tryptophan synthesis (Fukunaga *et al.* 1995, Qian *et al.* 2015). Thiocyanate in AS, APT, phosphoribose-o-aminobenzoate isomerase (PAI), indole-3-glycerol phosphate synthase (IGPS), TS, and tryptophan synthase β-under the catalysis of enzymes such as subunits (TSp), tryptophan is generated (Gierl and Frey 2001). AS is composed of the α and β subunits and is a complex enzyme that catalyzes the production of tryptophan in plant cells (Shen *et al.* 2020). In organisms, APT is part of a multifunctional protein that interacts with other enzymes involved in tryptophan biosynthesis (Hutter *et al.* 1986). TS is an α2β2-bifunctional pyridoxal 50-phosphate (PLP)-dependent enzyme that catalyzes the last two steps of tryptophan biosynthesis in bacteria, plants, and fungi. TS also promotes amino acid metabolism (Raboni *et al.* 2009). In this study, in the tryptophan synthesis pathway, the gene stacking lines (*o2o16wx*, *o2o16*, and *o2wx*) detected the up-regulated expression of genes encoding AS and APT, which promoted tryptophan synthesis. Different combinations of the *o2*, *o16*, and *wx* gene pyramiding lines detected that the genes encoding TS were up-regulated and promoted tryptophan synthesis.

The degradation of tryptophan in plants is mainly achieved through two pathways (Kasahara 2016, Mashiguchi *et al.* 2011, Stepanova *et al.* 2011). The first pathway represents the conversion of tryptophan to (indol-3-yl) acetate under the action of tryptophan decarboxylase (TDC) and IAAO; the second pathway represents the formation of (indol-3-yl) acetate, catalyzed by TAA and YUCCA. In previous studies, in the *wx* background, no DEGs were detected in the genotypes of *o2wx* and *o2o16wx* (Wang *et al.* 2019b, 2019c). Compared with the recurrent parent ‘CML530’, the genes encoding IAAO and YUCCA were down-regulated in the tryptophan degradation pathway in *o2o2o16o16* mutant (Wang *et al.* 2019a). In this study consistent with the above observations, *o2o16wx* detected that the gene encoding IAAO was down-regulated in ‘QCL8010_4’ (*o2o16*), and inhibited the degradation of tryptophan during the conversion of indole-3-chloroform to indoleacetate. The genes encoding YUCCA were down-regulated in ‘QCL8010_4’ (*o2o16*) and ‘QCL8010_5’ (*o2wx*) during the conversion of indolepyruvate to indoleacetate, and inhibited tryptophan degradation.

The process of maize starch biosynthesis is coordinated by a variety of starch synthases (Chen *et al.* 2012, Xiao *et al.* 2016). In this study (Fig. 6), during the conversion of α-D-Glc and β-D-F6p to 1,4α-Glc, the genes encoding HK, PG, AGPase and SS were up-regulated and promoted starch synthesis, while during the conversion of 1,4α-Glc to amylopectin, the gene encoding GBE was up-regulated and promoted the synthesis of amylopectin in *o2o16wx* and *o2wx* mutants. Finally, during the conversion of 1,4α-Glc to amylose, the gene encoding GBSS was up-regulated and promoted the synthesis of amylose in *o2o16* mutant.

**Fig. 8.**
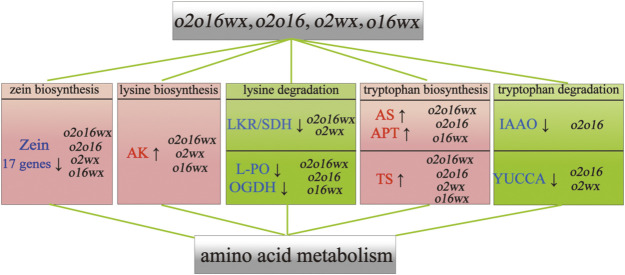
A proposed model of the regulatory network of ‘CML530’ (WT) corn following the introgression of the *o2*, *o16* and *wx*. ↑, up-regualted; ↓, down-regulated.

In this study, *o2*, *o16*, and *wx* were investigated in different combination types of gene pyramiding lines (Supplemental Table 8, Fig. 8). At stage of endosperm development (18DAP), RNA-Seq analysis revealed that the genes encoding L-PO, LKR/SDH, and OGDH were down-regulated during the conversion of Lys to acetyl-CoA by (S)2-aminoadipate-6-semialdehyde and L-2-aminoadipate, inhibiting the degradation of lysine. Meanwhile, in the tryptophan synthesis pathway, AS, APT, and TS were up-regulated and promoted the synthesis of tryptophan during the conversion of chorismate to anthranilate and N-(5-phosphoribosyl) anthranilic. In the case of the tryptophan degradation pathway, IAAO and YUCCA were down-regulated during the Try conversion (indol-3-yl) acetate process and inhibited tryptophan degradation. These results could help uncover the molecular mechanism in the increase lysine and tryptophan content, through pyramiding *o2*, *o16* and *wx* genes in maize.

## Author Contribution Statement

Data management, PZW and YLY; funding acquisition, RH and WW; project management, RH and WW; supervision, KWZ, LD, RH, WPY and WW; write the first draft, PZW and YLY; checking manuscripts, PZW, YLY, ZJM, KWZ, LD and WW.

## Supplementary Material

Supplemental Figures

Supplemental Table 1

Supplemental Table 2

Supplemental Table 3

Supplemental Table 4

Supplemental Table 5

Supplemental Table 6

Supplemental Table 7

Supplemental Table 8
