# Supplementary material for: Comparative transcriptome profiling reveals the mechanism of increasing lysine and tryptophan content through pyramiding opaque2, opaque16 and waxy1 genes in maize
Source: Breed Sci. 2024 Aug 14;74(4):311–23. doi: 10.1270/jsbbs.23051 (PMC11769590; doi:10.1270/jsbbs.23051)
Supplement: Supplementary file 1 — Supplemental Figures [file 74_311-s1.pdf]

Wu et al./Breeding Science
doi: 10.1270/jsbbs.23051

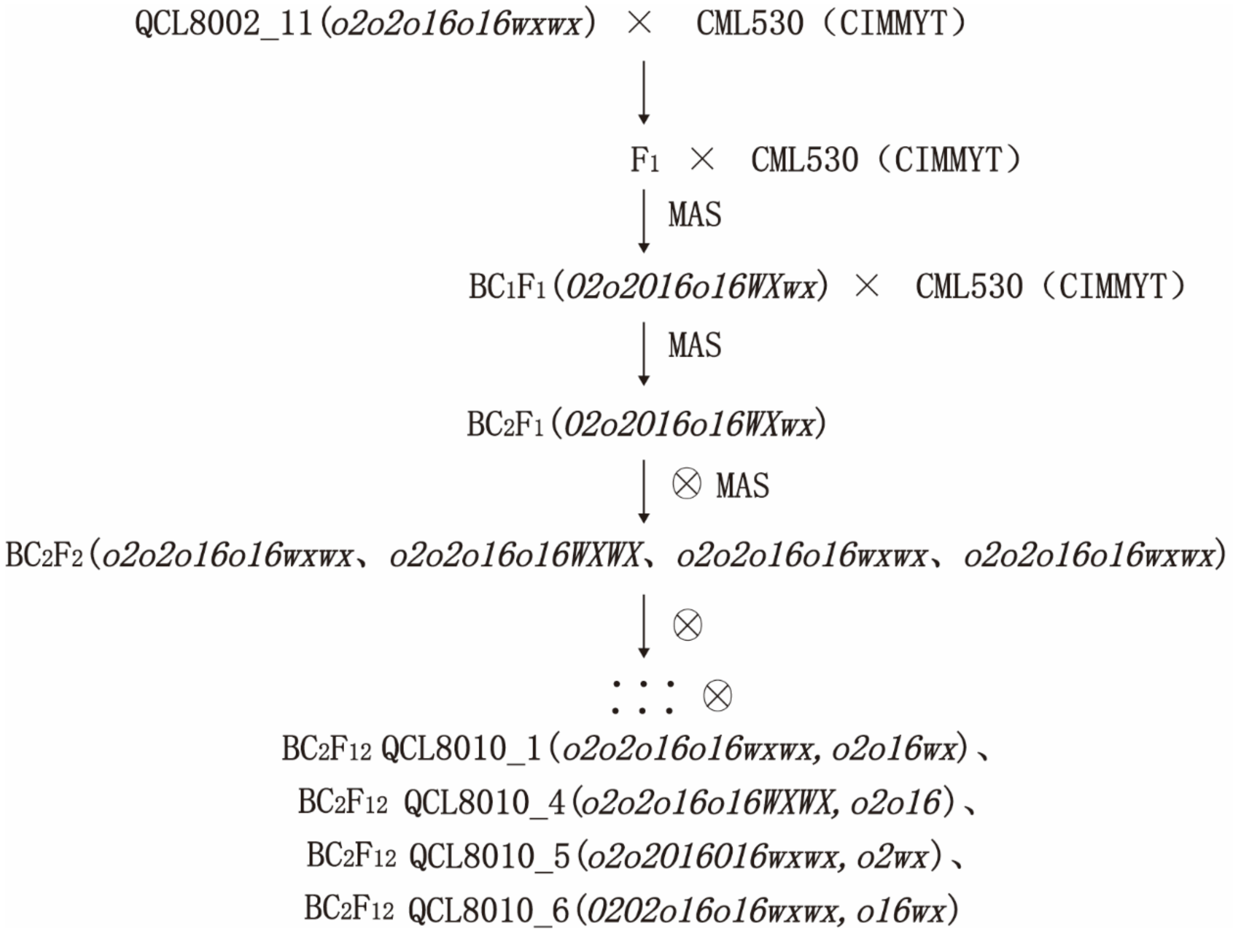

Supplemental Fig.1. A breeding flowchart adapted for the introgression of *o2*, *o16* and *wx* allele into the maize inbred CML530(WT).

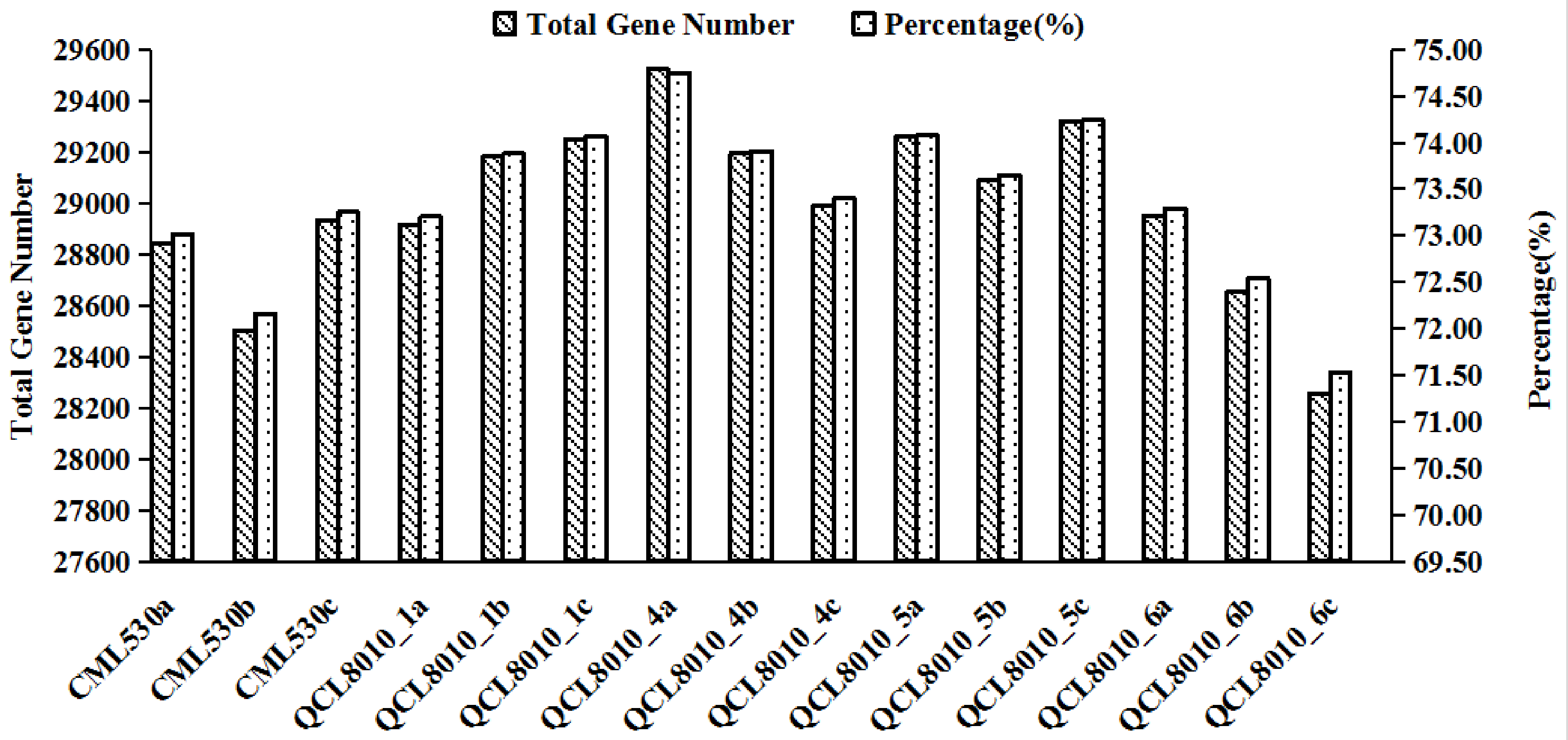

Supplemental Fig.2. The number and percentage of identified genes in CML530(WT) and gene pyramiding lines (*o2o16wx*, *o2o16*, *o2wx*, *o16wx*).

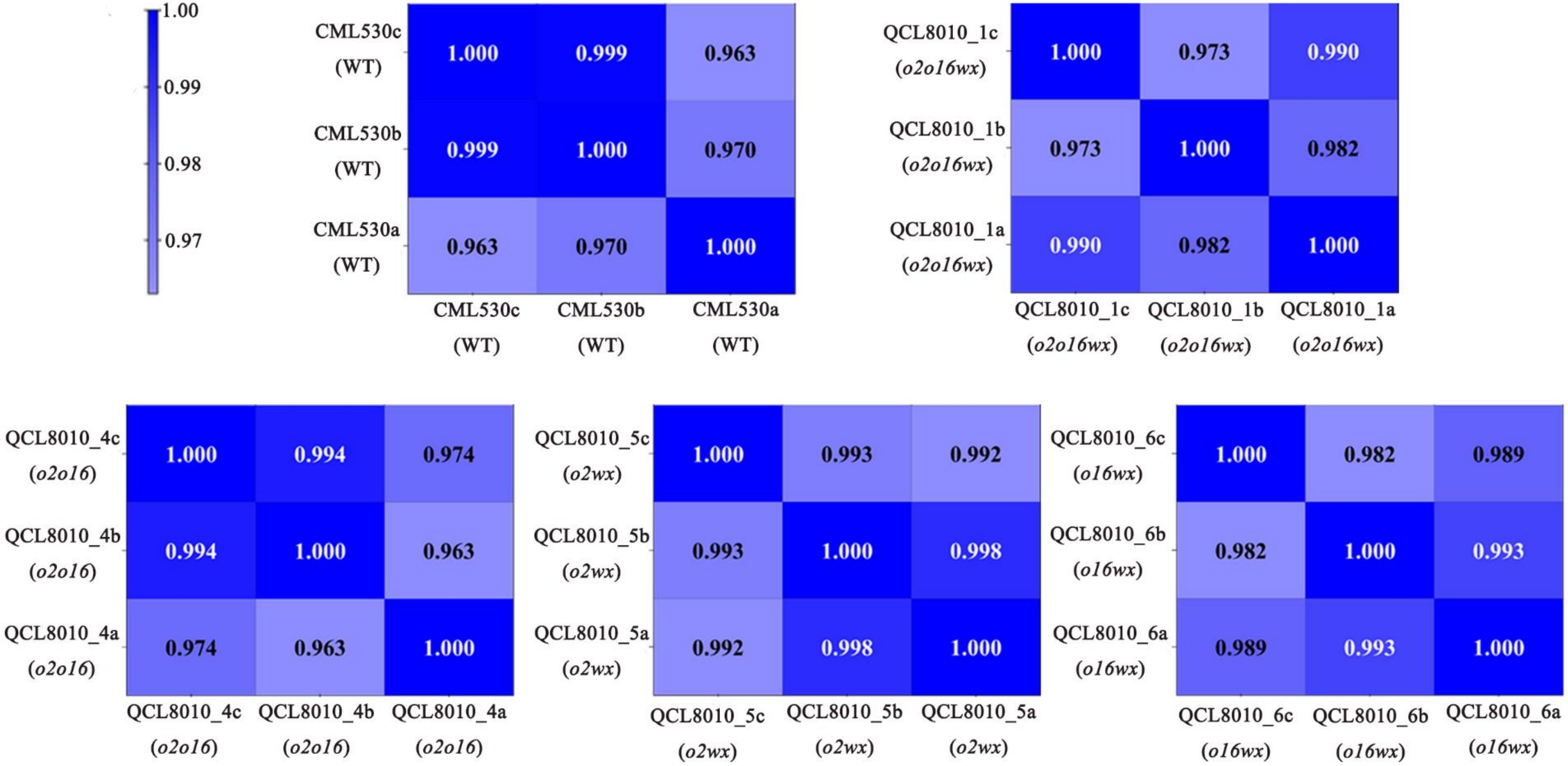

Supplemental Fig.3. The heatmap of correlation coefficient values acrossing samples. a, b, c represents three biological replicates.

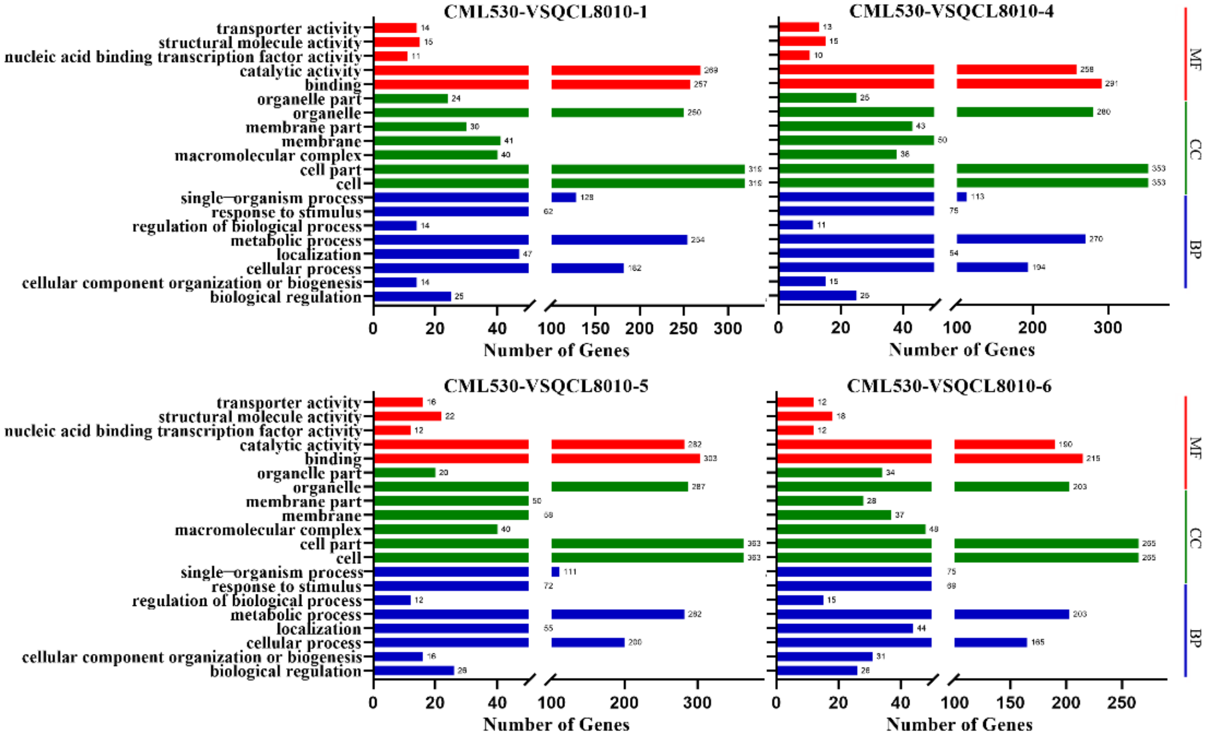

Supplemental Fig.4. GO functional annotation classification statistics of DEGs in CML530(WT) vs. QCL8010_1(*o2o16wx*), CML530(WT) vs. QCL8010_4(*o2o16*), CML530(WT) vs. QCL8010_5(*o2wx*) and CML530(WT) vs. QCL8010_6(*o16wx*).
